# Zebrafish: A Powerful Model for Genetics and Genomics

**DOI:** 10.3390/ijms24098169

**Published:** 2023-05-03

**Authors:** Seong-Kyu Choe, Cheol-Hee Kim

**Affiliations:** 1 Department of Microbiology, Wonkwang University School of Medicine, Iksan 54538, Republic of Korea; 2 Department of Biology, Chungnam National University, Daejeon 34134, Republic of Korea

Our understanding of fundamental biological mechanisms and the pathogenesis of human diseases has been greatly improved by studying the genetics and genomics of zebrafish [1]. The field of forward genetics has successfully identified functional genes that explain phenotypic presentations, contributing to the zebrafish model’s status as a key vertebrate model organism [2]. Recent advances in reverse genetics tools, including methods employing zinc finger nuclease, TALEN, and CRISPR-Cas9, have made zebrafish one of the most powerful model systems for breakthroughs in human diseases and provide the foundation for translational research [3].

In this Special Issue of “Zebrafish: a powerful model for genetics and genomics”, we present 16 research articles and reviews concerning zebrafish modeling in genetics and genomics that aid in understanding human diseases. Notable publications include three articles that illustrate the generation of novel transgenic zebrafish, which can be used in addressing important biological questions: (1) Mao et al. created a transgenic zebrafish to visualize adipocyte development, facilitating the characterization of white adipose tissues in vivo and the development of therapeutic interventions to treat metabolic diseases in humans; (2) Li et al. generated an animal system to monitor the in vivo dynamics of spatiotemporal calcium signaling useful for normal animal physiology as well as stressful and pathophysiological conditions; (3) Jeong et al. developed an optogenetic manipulation system for dissecting olfaction-related behaviors and their underlying neural circuitry.

In addition to transgenesis, two studies have shown advanced applications of CRISPR-Cas9 technology that could assist users in patient-specific gene tailoring as follows: (1) de Vrieze et al. revealed an improved strategy in developing knock-in zebrafish models; (2) Schellens et al. demonstrated the therapeutic potential using the exon skipping approach for modeling retinitis pigmentosa.

What makes this Special Issue more interesting is the addition of articles that each showed a novel gene knockout (KO) zebrafish that recapitulated clinical symptoms associated with specific human diseases. (1) Zhu et al. induced ciliopathy via a mutation in the *ift74* gene, developing gradual photoreceptor degeneration that is distinguishable from other previously published IFT-B mutants in zebrafish. (2) Berlingerio et al. demonstrated that cystinosis, a disease characterized by cystine accumulation in the lysosome resulting in nephropathy and vision problems in humans, was recapitulated in *ctns* gene mutant zebrafish. (3) Alexandro-Moreno et al. showed that primary congenital glaucoma, mainly associated with CYP1B1 gene mutations, was recapitulated in a *cyp1b1* KO zebrafish and displayed a novel pathway involving the dysregulated expression of extracellular matrix genes. (4) Kim et al. identified potential regulators responsible for vanishing white matter disease by performing a comparative proteome between *eif2b3* KO zebrafish and the WT control. These all show novel molecular processes that underlie disease etiology and, thus, could provide therapeutic clues for corresponding human diseases.

Additionally, several articles are included in this Special Issue to provide immediate benefit for researchers in regenerative biology, hematopoiesis, brain–skin communications, or environmental health. Ellman et al. described a tutorial paper for setting up an apex resection model to study heart regeneration in lab settings, providing an improved development model and assessing the heart’s capacity for regeneration using zebrafish. Huang et al. discovered that NELF (negative elongation factor), the promoter-proximal pausing controller of RNA polymerase II, is specifically involved in granulocyte development but not erythrocyte development, emphasizing the importance of NELF in regulating hematopoiesis. In addition, Ren et al. identified a biological mechanism that underlies environmental change, particularly acute cold resistance. Furthermore, Hong et al. developed a platform for screening MSH-related activity, identifying an effective chemical treatment for autism spectrum disorder.

Finally, three review articles were included in the Special Issue in order to highlight the current status of disease modeling using zebrafish and the underlying molecular pathogenesis. Specific topics include hematopoiesis, rare neurological disorders, and Axenfeld–Rieger syndrome (ARS): (1) Zhang et al. summarized a comprehensive review on erythropoiesis using zebrafish models to explore human anemias; (2) Son et al. described the disease modeling of rare neurological disorders, focusing on zebrafish models; (3) French C.R. wrote a review on the development of ARS induced by mutations in *FOXC1* and *PITX2* and discussed their analyses on the utility of zebrafish with the corresponding gene mutations for dissecting potential molecular mechanisms and therapeutic approaches.

In summary, this Special Issue is a collection of 16 relevant articles that support the use of zebrafish modeling in genetics and genomics for treating human diseases. To further boost the benefits for the communities of researchers who use zebrafish models, we are accepting original research papers or review articles for the 2.0 version with a setup similar to this Special Issue: “Zebrafish: A Powerful Model for Genetics and Genomics 2.0”.

## Data Availability

There is no data associated with the study.
